# Dispersion of Milk

**Published:** 1857-04

**Authors:** 


*Dispersion of Milk.*

M. Coutenot has found the expressed oil hemp seed an admirable means for the rapid dispersion of the milk, employing it in abundant warm frictions, and then leaving the breast covered with wadding soaked in it. It is of no effect where inflammatory action, consequent upon ingorgement, has set in.—*Union Ned.*

